# HIV-1 Genomes Are Enriched in Memory CD4^+^ T-Cells with Short Half-Lives

**DOI:** 10.1128/mBio.02447-21

**Published:** 2021-09-21

**Authors:** Vincent Morcilla, Charline Bacchus-Souffan, Katie Fisher, Bethany A. Horsburgh, Bonnie Hiener, Xiao Qian Wang, Timothy E. Schlub, Mark Fitch, Rebecca Hoh, Frederick M. Hecht, Jeffrey N. Martin, Steven G. Deeks, Marc K. Hellerstein, Joseph M. McCune, Peter W. Hunt, Sarah Palmer

**Affiliations:** a Centre for Virus Research, The Westmead Institute for Medical Research, The University of Sydney, Sydney, New South Wales, Australia; b Division of Experimental Medicine, Department of Medicine, University of California San Francisco, San Francisco, California, USA; c Sydney Medical School, Westmead Clinical School, Faculty of Medicine and Health, The University of Sydney, Sydney, New South Wales, Australia; d Sydney School of Public Health, Faculty of Medicine and Health, The University of Sydney, Sydney, New South Wales, Australia; e Department of Nutritional Sciences and Toxicology, University of California Berkeley, Berkeley, California, USA; f Division of HIV, Infectious Diseases and Global Medicine, Department of Medicine, Zuckerberg San Francisco General Hospital, University of California San Francisco, San Francisco, California, USA; g Global Health Innovative Technology Solutions/HIV Frontiers, Bill & Melinda Gates Foundation, Seattle, Washington, USA; Columbia University Medical College

**Keywords:** cell proliferation, cellular half-life, human immunodeficiency virus, persistence

## Abstract

Future HIV-1 curative therapies require a thorough understanding of the distribution of genetically-intact HIV-1 within T-cell subsets during antiretroviral therapy (ART) and the cellular mechanisms that maintain this reservoir. Therefore, we sequenced near-full-length HIV-1 genomes and identified genetically-intact and genetically-defective genomes from resting naive, stem-cell memory, central memory, transitional memory, effector memory, and terminally-differentiated CD4^+^ T-cells with known cellular half-lives from 11 participants on ART. We find that a higher infection frequency with any HIV-1 genome was significantly associated with a shorter cellular half-life, such as transitional and effector memory cells. A similar enrichment of genetically-intact provirus was observed in these cells with relatively shorter half-lives. We found that effector memory and terminally-differentiated cells also had significantly higher levels of expansions of genetically-identical sequences, while only transitional and effector memory cells contained genetically-intact proviruses that were part of a cluster of identical sequences. Expansions of identical sequences were used to infer cellular proliferation from clonal expansion. Altogether, this indicates that specific cellular mechanisms such as short half-life and proliferative potential contribute to the persistence of genetically-intact HIV-1.

## INTRODUCTION

One of the major barriers to an HIV-1 cure is the persistence of replication-competent HIV-1 in memory CD4^+^ T-cells during antiretroviral therapy (ART) (1–3). Although ART reduces viremia to low levels, the virus persists in cellular reservoirs, and treatment cessation results in the resumption of viral replication (4, 5). Understanding the cellular mechanisms that contribute to the persistence of HIV-1 in cells during therapy will be vital to developing future curative strategies.

Previous studies have demonstrated that the number of HIV-1-infected cells remains remarkably stable during ART (3, 6), despite cell death and a lack of ongoing viral replication (7). Cellular proliferation has been found to contribute to the persistence of HIV-1 in memory T-cells (5, 6, 8–18), and this can occur through clonal expansion after exposure to cognate antigens, through binding of homeostatic cytokines, or as a result of the proviral integration site (19–22). However, these proliferative events are influenced by the diversity of the CD4^+^ T-cell compartment. T-cell subsets exist across a gradient of differentiation, from less differentiated naive (NV), stem-cell memory (SCM), and central memory (CM) populations to more differentiated transitional memory (TM), effector memory (EM), and terminally-differentiated (TD) cells (19, 23). This linear differentiation gradient reflects an increasing potential of T-cells to proliferate in response to cognate antigens.

Recent studies have shown that genetically-intact HIV-1 proviruses are unequally distributed across CD4^+^ T-cell subsets and anatomical compartments (17, 24, 25). We have previously observed higher levels of genetically-intact provirus in EM T-cells and cells expressing the activation marker human leukocyte antigen-antigen D related (HLA-DR) cells during long-term ART (24, 25). Analysis of the unique characteristics of each T-cell subset may inform how genetically-intact HIV-1 persists within these cells during ART. In particular, markers for CD4^+^ T-cell subsets at various maturation stages (26, 27) provide the opportunity to quantify the turnover of these discrete subsets and examine the relationship between cellular replacement rates and HIV-1 persistence during ART (28).

The timing of ART initiation may influence the level and genetic characteristics of persistent HIV-1 in treated individuals (6, 10). Early ART initiation limits the seeding of HIV-1 infection; however, studies have shown that this has little effect on HIV-1 disease outcome once ART is interrupted (29–33). How timing of ART initiation affects the level and distribution of genetically-intact proviruses in participants on effective ART is unclear and requires investigation.

In this study, we employed the full-length individual proviral sequencing (FLIPS) assay to identify genetically-intact HIV-1 proviral sequences within peripheral blood resting CD4^+^ T-cell subsets with known half-lives (24, 28). We found that T-cells with a relatively shorter half-life (i.e., EM and TM cells) have a higher proviral infection frequency. Importantly, this finding is associated with the turnover rate of a cell, suggesting that cellular replacement rates of specific cell subsets play a role in maintaining persistent HIV-1 across CD4^+^ T-cells. The half-life or replacement rate of a cell subset can include cells leaving or entering the compartment due to cell death, cellular differentiation, or migration into a different anatomical site. We will continue to refer to these collectively as cellular half-lives. Furthermore, we found that cells with shorter half-lives have higher levels of both genetically-identical sequences and genetically-intact identical sequence expansions, which suggests that cellular proliferation is an important mechanism for maintaining persistent HIV-1 in the peripheral blood during ART. We found no significant difference in the HIV-1 infection frequency and levels of genetically-intact HIV-1 provirus as a function of when ART was initiated. This study provides evidence that cellular mechanisms such as half-life and proliferation play important roles in maintaining genetically-intact HIV-1 during ART.

## RESULTS

**FIG 1:**
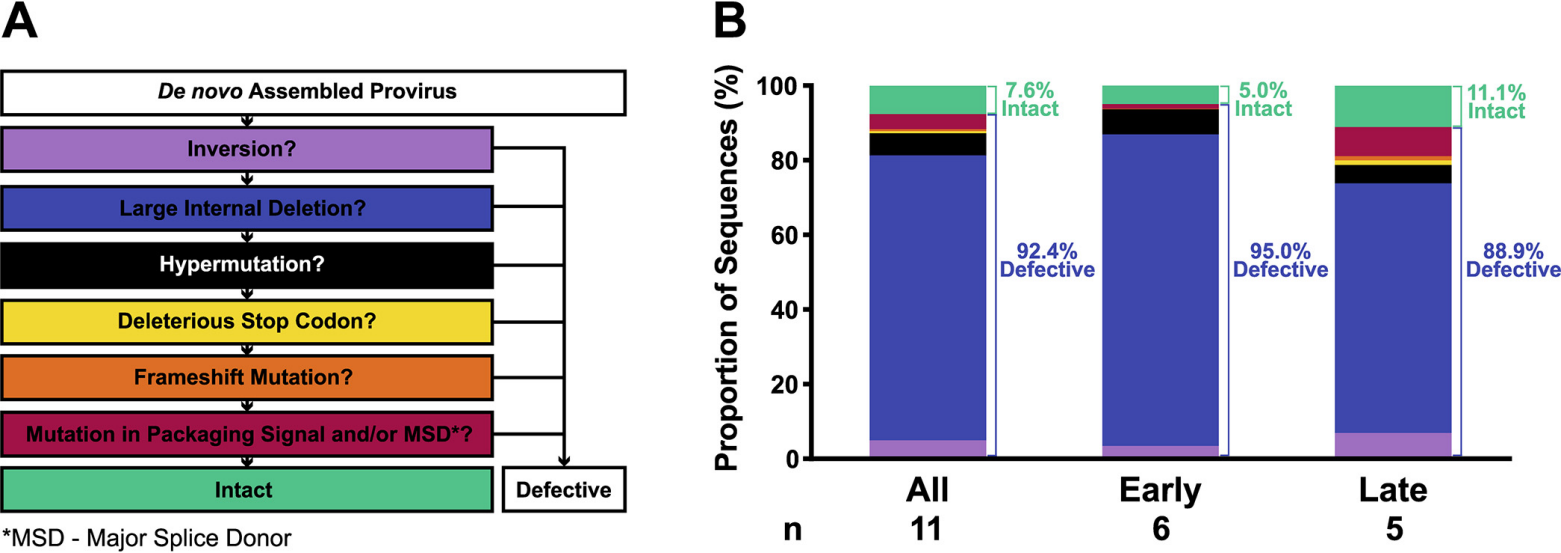
Proportion of defective and intact viral DNA sequences isolated from each ART treatment group. (A) Sequence classification pipeline. Sequences were classified by a process of elimination that identified the major defects within each sequence. (B) Characteristics of sequences are colored the same as those for panel A. Number of participants is noted under each group.

We obtained peripheral blood resting CD4^+^ T-cell subsets (NV, SCM, CM, TM, EM, and TD) from large blood draws in 11 participants, 6 who initiated ART <6 months after infection (early group) and 5 who initiated ART >6 months after infection (late group) (Table 1). The fractional replacement rates (that is, the proportion of cells replaced per day) of these cell subsets were estimated from *in vivo* deuterium labeling (28). This was used to calculate cellular half-lives for each cell subset in each participant (Table 2). The fractional replacement rates calculated for each cell subset was similar between 24 ART-suppressed HIV-1-infected participants and 6 uninfected participants, although the size of the cellular pool for each cell subset was lower in the ART-suppressed HIV-1-infected participants compared to uninfected participants (28). The FLIPS assay (24) was employed to sequence near-full-length HIV-1 genomes at the single-genome level. HIV-1 genomes were considered genetically-intact if they lacked genetic defects such as inversions, large internal deletions, hypermutation, deleterious stop codons, frameshifts or mutations in the packaging signal and/or major splice donor (MSD) site (*cis-*acting region) (Fig. 1A) (24).

**TABLE 1:** Participant demographics

Group and SCOPE PID	Sex	Age	Viral load^*a*^ (copies/ml)	CD4^+^ T-cell count^*a*^ (cells/μl)	Time of infection before initiation of therapy (mo)	ART duration^*a*^ (yr)	ART duration^*a*^ (classification)
Early							
2647	Male	33	<40	532	4.5	3.4	Short
2531	Male	51	<40	1,163	1.9	3.4	Short
2664	Male	46	<40	637	4.1	2.7	Short
2606	Male	29	<40	787	1.7	3.5	Short
2454	Male	35	<40	513	0.7	7.1	Long
2661	Male	54	<40	739	3.4	12.9	Long
Late							
1408	Male	31	<40	637	45.5	3.1	Short
3632	Male	31	<40	902	20.8	1.8	Short
1756	Male	29	<40	582	6.8	4.1	Short
2274	Male	54	<40	486	13.1	11.8	Long
2208	Male	64	<40	437	114.5	7.1	Long

a At the time of sampling.

**TABLE 2:** Cellular half-lives for each subset and participant

Group and SCOPE PID	Half-life (days)					
	NV	SCM	CM	TM	EM	TD
Early						
2647	488	160	103	94	88	165
2531	839	258	149	111	82	274
2664	2,484	184	202	143	151	536
2606	1,451	138	145	114	156	296
2454	2,146	139	135	105	108	245
2661	2,361	266	430	261	174	385
Median	1,799	172	147	113	130	285
Late						
1408	1,747	153	144	95	64	291
3632	766	126	106	74	71	203
1756	1,107	165	93	77	52	244
2274	904	99	135	120	133	221
2208	557	89	128	97	98	238
Median	904	126	128	95	71	238

### Proviral characteristics.

A total of 1,277 viral sequences were isolated from the 11 participant samples. While the majority of these sequences (92.4%) were defective, we were able to identify 97 genetically-intact proviral sequences (7.6%) (Fig. 1B, Table 3). Of the 1,277 viral sequences, 727 were isolated for the early group, while 550 were isolated for the late group. In both groups, the majority of sequences were found to contain large internal deletions, comprising 95.0% of sequences in the early group and 88.9% of sequences in the late group. For the early and late groups, the proportion of hypermutated sequences was 6.7% and 4.9%, respectively (*P* = 0.79, mixed-effects logistic regression). We identified sequences in the late group that were defective due to a deleterious stop codon caused by a single-nucleotide polymorphism (1.3% of total sequences), and this type of defect was not found in the early group. We found 7.8% of the defective sequences from the late group contained a defect in the *cis*-acting region, whereas only 1.2% of these defects were found in the early group (*P* = 0.034, mixed-effects logistic regression). For the early and late groups, 5.0% and 11.1% of the total sequences were genetically-intact, respectively (*P* = 0.27, mixed-effects logistic regression).

**TABLE 3:** Number of cells and sequences obtained for each subset and participant

Group and SCOPE PID	No. of sequences and CD4^+^ T-cells used for analysis																	
	NV			SCM			CM			TM			EM			TD		
	Cells analyzed	Defective	Intact	Cells analyzed	Defective	Intact	Cells analyzed	Defective	Intact	Cells analyzed	Defective	Intact	Cells analyzed	Defective	Intact	Cells analyzed	Defective	Intact
Early																		
2647	2,008,627	20	1	104,356	2	0	606,738	49	2	488,060	35	0	557,995	46	2	355,176	38	1
2531	846,802	2	1	505,614	11	1	845,044	26	0	369,349	33	1	241,380	37	0	589,001	33	2
2664	2,460,392	12	0	NA	NA	NA	1,124,804	42	0	750,758	24	1	285,373	56	0	350,372	39	0
2606	2,377,831	NA	NA	NA	NA	NA	3,074,632	10	0	2,709,673	34	1	1,419,446	21	0	794,481	2	0
2454	944,567	7	0	NA	NA	NA	822,853	34	0	606,953	42	1	370,087	26	0	NA	NA	NA
2661	1,312,753	NA	NA	NA	NA	NA	1,005,587	1	0	978,764	0	1	1,125,278	9	21	NA	NA	NA
Late																		
1408	2,008,675	1	0	NA	NA	NA	666,109	39	0	288,519	46	0	196,068	20	6	231,663	8	0
3632	1,972,938	6	2	155,591	3	0	844,189	14	0	1,200,376	31	2	409,945	35	0	677,269	3	0
1756	2,860,172	NA	NA	131,518	2	0	1,024,396	15	0	997,705	19	15	679,114	18	22	175,675	NA	NA
2274	342,384	NA	NA	NA	NA	NA	1,107,273	25	1	825,005	43	0	350,637	25	10	NA	NA	NA
2208	1,010,249	36	0	NA	NA	NA	367,777	35	1	419,586	31	2	267,978	34	0	248,955	NA	NA

### HIV-1 is enriched in memory CD4^+^ T-cells with short half-lives.

**FIG 2:**
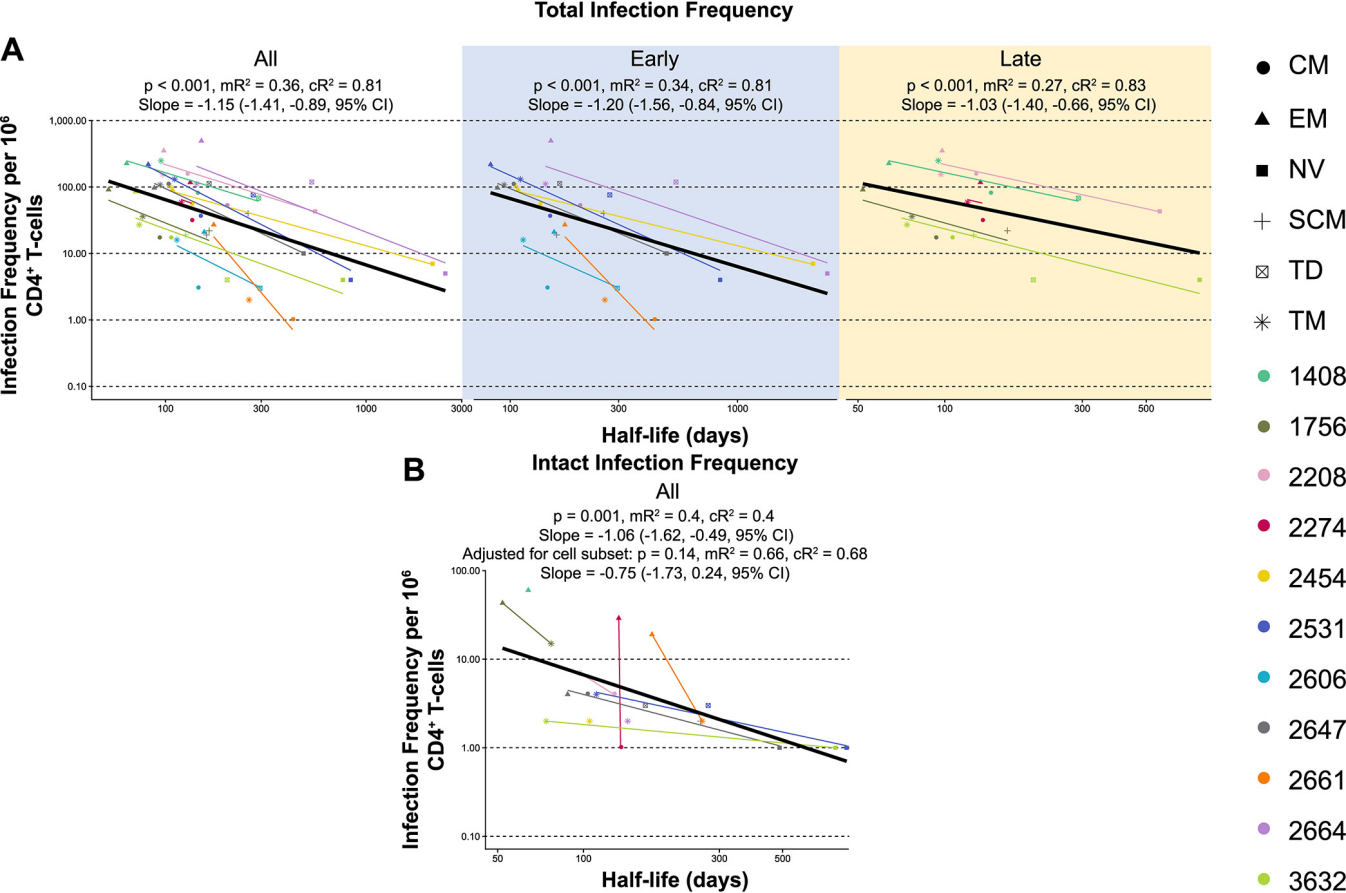
Infection frequency versus cellular half-life. (A) Mixed-effects modeling of total infection frequency per 10^6^ cells versus cellular half-life (days) for all participants and early and late ART treatment groups. (B) Mixed-effects modeling of intact infection frequency per 10^6^ cells versus cellular half-life (days) for all participants. The black line depicts simple regression analysis. *P* values and *R*^2^ values can be found for all participants and each ART treatment group. Two *R*^2^ values are calculated for this model, m*R*^2^ (marginal) and c*R*^2^ (conditional), which are similar to a linear regression comparing the relationship between half-life and infection frequency for individual participants or the population as a whole. Marginal *R*^2^ is calculated using all the data points on the graph regardless of participant, whereas the conditional *R*^2^ accounts for participant variability. The slopes and 95% CIs are reported for the population-wide trend.

For each participant, we investigated whether the measured cellular half-lives of each CD4^+^ T-cell subset (Table 2) (28) were associated with HIV-1 infection frequencies. HIV-1 infection frequency was calculated for each participant and CD4^+^ T-cell subset (NV, SCM, CM, TM, EM, and TD) using the total number of sequences (intact or defective) amplified and the total number of cells collected for each subset. Cellular half-lives across all participants (Table 2) ranged from the longest in the NV subset (median, 1,107 days; interquartile range [IQR], 1,144 days) to the shortest in the EM subset (median, 98 days; IQR, 65.5 days). To determine the role of individual participant factors on infection frequencies, conditional and marginal *R*^2^ (c*R*^2^ and m*R*^2^, respectively) values were calculated for the association between cellular half-lives and HIV-1 infection frequencies. These indicate the amount of variability of infection frequency that can be accounted for by cellular half-life. The m*R*^2^ is the variability accounted for by the population-wide trend in half-life (Fig. 2, black lines). The c*R*^2^ is the variability accounted for by the trend in half-life after adjusting for individual participant effects (Fig. 2, individual participant colored lines).

Analysis of all participants demonstrated strong evidence for an association between cells with shorter half-lives and higher proviral loads (*P* < 0.001, slope = −1.15 [−1.41, −0.89, 95% confidence interval, or CI], m*R*^2^ = 0.36, c*R*^2^ = 0.81; Fig. 2A, mixed-effects linear regression). The trend between half-life and infection frequency is also present when the participants were divided into the separate early and late ART treatment groups (*P* < 0.001 for both, m*R*^2^ = 0.34 and c*R*^2^ = 0.81 for early, m*R*^2^ = 0.27 and c*R*^2^ = 0.83 for late; Fig. 2A, mixed-effects linear regression). When time to ART initiation (Table 1) was treated as a continuous variable, the trend between half-life and infection frequency remained after adjusting for time to ART initiation (data not shown).

For all participants, to assess whether the association between total infection frequency and cellular half-life is independent of other cellular subset effects, we next measured the association between HIV-1 infection frequency and cellular half-life while adjusting for cellular subset. We found that higher infection frequencies were still associated with shorter cellular half-lives (*P* = 0.026, slope = −0.86 [−1.63, −0.10, 95% CI], m*R*^2^ = 0.39, c*R*^2^ = 0.87). To investigate whether this relationship was driven by NV T-cells that exhibit both long half-lives and low infection frequencies, we repeated the analysis without these cells (see Table S1 in the supplemental material). This regression analysis without NV T-cells also revealed strong evidence that a cell containing an HIV-1 genome is more likely to have a shorter half-life (*P* < 0.001 without cellular subset adjustment, *P* = 0.043 with cellular subset adjustment). Together, this provides strong evidence that when accounting for participant variability, subsets with higher HIV-1 infection frequencies have shorter half-lives.

10.1128/mBio.02447-21.1 TABLE S1 Linear regression analysis excluding NV T-cells. Download Table S1, DOCX file, 0.01 MB. Copyright © 2021 Morcilla et al. 2021 Morcilla et al. https://creativecommons.org/licenses/by/4.0/ This content is distributed under the terms of the Creative Commons Attribution 4.0 International license.

We next investigated the association between cellular half-life and the frequency of genetically-intact HIV-1 sequences. We found strong evidence that a cell containing a genetically-intact HIV-1 genome is more likely to have a shorter half-life (*P* = 0.001, slope = −1.06 [−1.62, −0.49, 95% CI], m*R*^2^ = 0.4, c*R*^2^ = 0.4) (Fig. 2B). This relationship exists in both the early and late groups separately (*P* = 0.016 and *P* = 0.032, respectively) (Fig. S1) and remains when NV T-cells were removed from the regression analysis (*P* = 0.018) (Table S1). We also investigated evidence for a relationship between genetically-intact infection frequency and cellular half-life after adjusting the data for cellular subset and found this trend was no longer statistically significant (*P* = 0.14, slope = −0.75 [−1.73, 0.24, 95% CI], m*R*^2^ = 0.66, c*R*^2^ = 0.68). We note that the low number of genetically-intact sequences isolated from each cellular subset (Table 3) means that this comparison has low statistical power.

10.1128/mBio.02447-21.3 FIG S1 Intact infection frequency versus cellular half-life for early and late groups. Mixed-effects modelling of intact infection frequency per 10^6^ cells versus cellular half-life (days) for early and late ART treatment groups was used. The black line depicts simple regression analysis. *P* values and *R*^2^ values can be found for each analysis. Two *R*^2^ values are calculated for this model, c*R*^2^ (conditional) and m*R*^2^ (marginal), which are similar to a linear regression comparing the relationship between half-life and infection frequency for individual participants or the population as a whole. Marginal *R*^2^ is calculated using all the data points on the graph regardless of participant, whereas the conditional *R*^2^ accounts for participant variability. Slopes and 95% CIs are reported for the population-wide trends. Download FIG S1, TIF file, 1 MB. Copyright © 2021 Morcilla et al. 2021 Morcilla et al. https://creativecommons.org/licenses/by/4.0/ This content is distributed under the terms of the Creative Commons Attribution 4.0 International license.

### Expansions of identical sequences were found in more differentiated CD4^+^ T-cells.

Cellular proliferation has been shown to contribute to the persistence of HIV-1 proviruses in CD4^+^ T-cells (5, 6, 8–18). The presence of genetically-identical proviruses is likely to be indicative of cellular proliferation, as the error rate of the HIV-1 reverse transcriptase limits the transcription of identical proviruses by *de novo* replication (34). As such, when phylogenetic analyses were conducted for the HIV-1 sequences obtained from each participant sample, we inferred host cell proliferation through the presence of clusters consisting of ≥2 identical proviral sequences or expansions of identical sequences (EIS) (Fig. S2 and S3).

10.1128/mBio.02447-21.4 FIG S2 Phylogenetic tree of HIV-1 sequences from the early ART initiation group (participants 2647, 2531, 2664, 2606, 2661, and 2454). For each participant, a maximum likelihood phylogenetic tree is found in the center with subsets color coded: NV (blue), SCM (yellow), CM (green), TM (orange), EM (red), and TD (purple). In the inner concentric circle, sequences colored cyan are part of an EIS. The shading depicts the particular cell subset that forms the EIS, and grey shading reveals an EIS formed by multiple cell subsets. The outer concentric circle depicts full-length intact sequences by color corresponding to the cell subset containing that sequence. Sequence lengths are depicted in the outer ring. Download FIG S2, TIF file, 2.9 MB. Copyright © 2021 Morcilla et al. 2021 Morcilla et al. https://creativecommons.org/licenses/by/4.0/ This content is distributed under the terms of the Creative Commons Attribution 4.0 International license.

10.1128/mBio.02447-21.5 FIG S3 Phylogenetic tree of HIV-1 sequences from the late ART initiation group (participants 1408, 3632, 1756, 2274, and 2208). For each participant, a maximum likelihood phylogenetic tree is found in the center with subsets color coded: NV (blue), SCM (yellow), CM (green), TM (orange), EM (red), and TD (purple). In the inner concentric circle, sequences colored cyan are part of an EIS. The shading depicts the particular cell subset that forms the EIS, and grey shading reveals an EIS formed by multiple cell subsets. The outer concentric circle depicts full-length intact sequences by color corresponding to the cell subset containing that sequence. Sequence lengths are depicted in the outer ring. Download FIG S3, TIF file, 2.5 MB. Copyright © 2021 Morcilla et al. 2021 Morcilla et al. https://creativecommons.org/licenses/by/4.0/ This content is distributed under the terms of the Creative Commons Attribution 4.0 International license.

**FIG 3:**
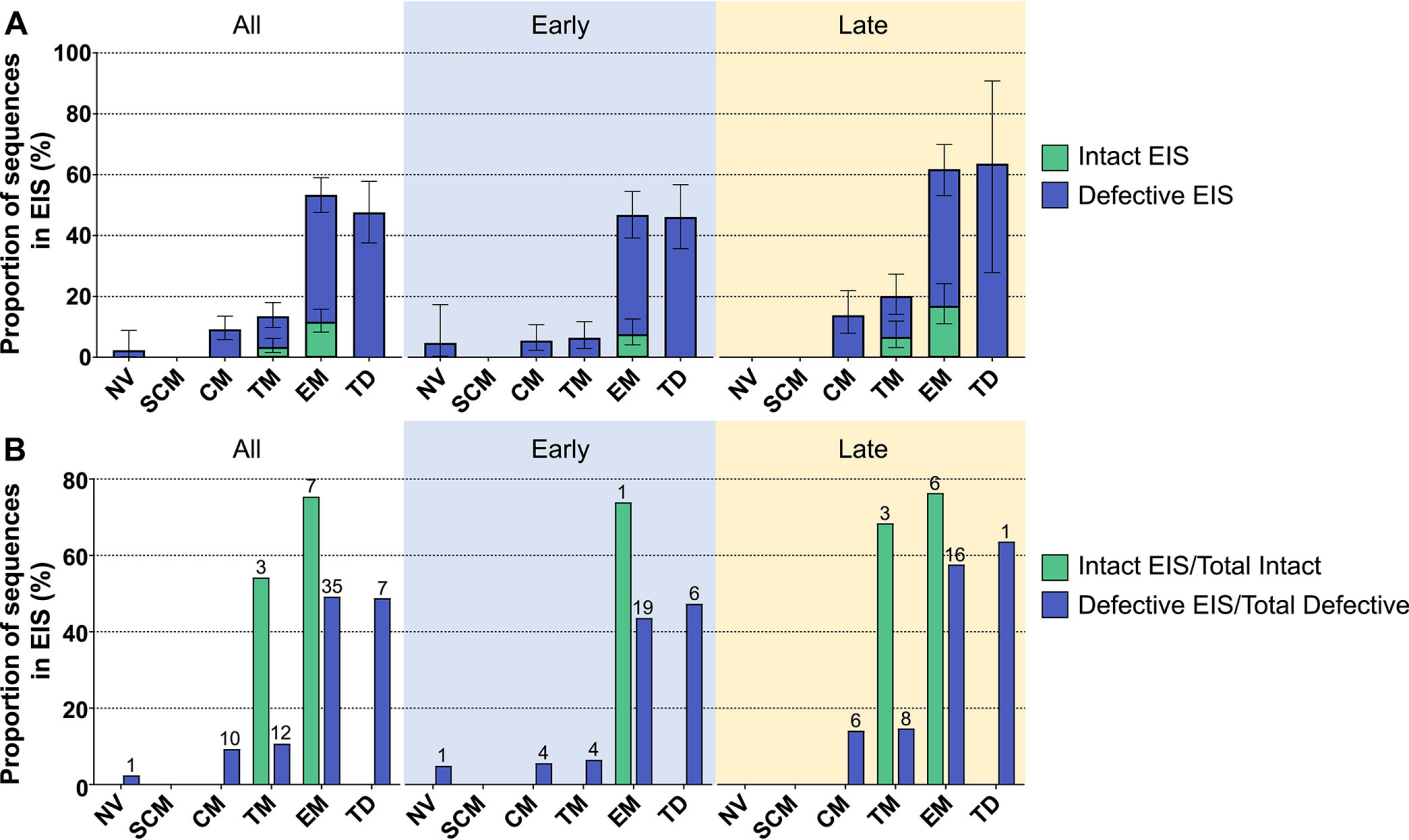
Expansions of identical sequences for each ART treatment group. (A) Proportion of all sequences, either defective (blue) or intact (green), in an EIS in each CD4^+^ T-cell subset for all participants and early and late ART treatment groups. Data represented as means ± 95% CI. (B) The proportion of intact (green) and defective (blue) sequences that are also part of an EIS. The number of clusters that contribute to the pool of identical sequences is noted above the bars.

Sequences that were part of an EIS represented 27% of all sequences. The proportion of sequences belonging to an EIS was found to be highest in EM (53% of all EM sequences) and TD (48% of all TD sequences) compared to the other cell subsets studied (Fig. 3A) (*P *< 0.001, mixed-effects logistic regression). We note here that the proportion of sequences that were part of an EIS in the TD subset may have been reduced by potential contamination of this subset with NV T-cells. There was no difference in the overall proportion of sequences in an EIS between the early (24%) and late (31%) groups. However, when we compared the proportion of EIS between subsets, we observed TM to be significantly higher in the late group than the early group (*P* < 0.001, mixed-effects logistic regression). No other T-cell subset showed a statistically significant difference in the proportion of sequences belonging to an EIS between ART treatment groups (Table S2).

10.1128/mBio.02447-21.2 TABLE S2 Comparisons between early and late ART treatment groups (mixed-effects logistic regression). Download Table S2, DOCX file, 0.01 MB. Copyright © 2021 Morcilla et al. 2021 Morcilla et al. https://creativecommons.org/licenses/by/4.0/ This content is distributed under the terms of the Creative Commons Attribution 4.0 International license.

Genetically-intact sequences that were part of an EIS made up 4.6% of total sequences from the 11 participants. Only TM and EM cells contained genetically-intact sequences within an EIS. The proportion of genetically-intact sequences in an EIS was 3.6% and 12% of total sequences in TM and EM, respectively (Fig. 3A). There was no evidence for a difference in the overall proportion of genetically-intact sequences that were part of an EIS between the early and late groups (*P* = 0.23, mixed-effects logistic regression). Within each cell subset, there was no evidence for a difference in the number of genetically-intact sequences belonging to an EIS between the early and late groups (*P* > 0.2 for all, mixed-effects logistic regression) (Table S2).

We also assessed the contribution that sequences in an EIS have toward the total number of either genetically-intact or defective proviruses (Fig. 3B) (i.e., the number of sequences that were genetically-intact and part of an EIS over the total number of genetically-intact sequences and the number of sequences that were defective and part of an EIS over the total number of defective sequences). This was compared between cell subsets as well as between the early and late treatment groups. Although only 27% of all sequences were part of an EIS within the cells from the 11 participants, we found that 61% of all genetically-intact sequences belonged to an EIS. This finding suggests that genetically-intact sequences were disproportionately found within an EIS. Interestingly, 68% of the genetically-intact sequences in the TM subset were part of an EIS in the late group, while no genetically-intact sequences in an EIS were identified in the TM subset in the early group. However, this high proportion of genetically-intact identical sequences within the late group was formed by 3 clusters isolated from 1 participant, indicating that this result was driven by a single participant. The proportion of genetically-intact sequences in an EIS in EM was similar in the early (74%) and late groups (76%); however, this was also driven by a small number of clusters that contributed a large number of identical sequences. For EM, the proportion of defective sequences in an EIS was higher in the late group (58%) than the early group (44%) (*P* = 0.014, Fisher exact test). In the TD subset, we found the proportion of defective sequences in an EIS was 64% in the late group and 47% in the early group (*P* = 0.36, Fisher exact test). For the late group, this result came from one cluster of identical sequences from one participant.

### Genetically-intact proviruses are unequally distributed in CD4^+^ T-cell subsets.

To determine whether genetically-intact proviruses would be concentrated in cell subsets with the shortest half-life (i.e., EM and TM), we assessed the frequency of HIV-1-infected cells (i.e., cells containing any HIV-1 genome, whether intact or defective) within resting NV, SCM, CM, TM, EM, and TD to determine the distribution of total and genetically-intact provirus within these T-cell subsets. Recent studies have investigated the distribution of genetically-intact HIV-1 provirus in NV, CM, TM, and EM subsets (24, 25), but the distribution of genetically-intact provirus in those that are resting as well as SCM and TD subsets has not been determined.

**FIG 4:**
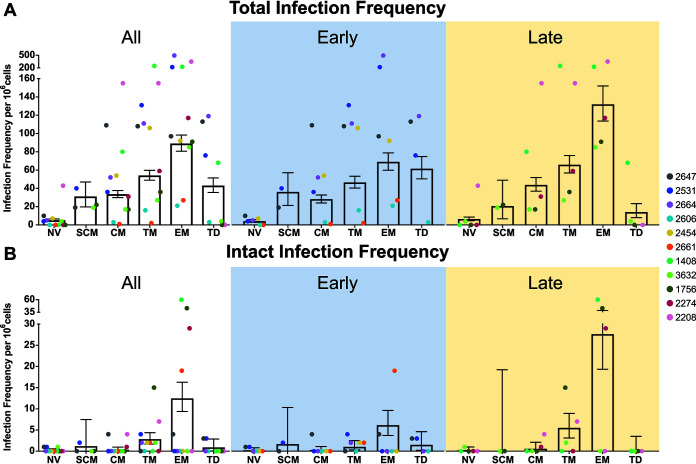
Infection frequency for each cell subset in each ART treatment group. (A) Total infection frequency per 10^6^ cells for each CD4^+^ T-cell subset for all participants (*n* = 11) and each ART treatment group, early (*n* = 6) and late (*n* = 5). (B) Intact infection frequency per 10^6^ cells for each CD4^+^ T-cell subset for all participants (*n* = 11) and each ART treatment group, early (*n* = 6) and late (*n* = 5). Data represented as mean ± 95% CI.

We first investigated the distribution of HIV-1-positive cells in each cell subset (Fig. 4A). We found strong evidence for a difference in the number of HIV-1-positive cells across the cell subsets (*P* < 0.001, mixed-effects logistic regression). We observed the order of this infection frequency to be EM>TM>TD/CM/SCM>NV (*P* < 0.005, mixed-effects logistic regression). There was a large range of infection frequencies measured between these subsets, with EM having an average of 89 proviruses per 10^6^ cells and NV having an average of 5 proviruses per 10^6^ cells (Fig. 4A). Within each cell subset, there was no evidence for a difference in estimated total infection frequency between the early and late groups (Table S2). When time to ART initiation (Table 1) was treated as a continuous variable, there was weak evidence for a positive association between total HIV-1 infection frequency and time to ART initiation in NV, CM, and EM, with the late treatment participants having a higher frequency of HIV-1 genomes in these cell subsets (0.05 < *P* = 0.10, mixed-effects logistic regression; Table S2).

We investigated the distribution of intact HIV-1 provirus in each of the CD4^+^ T-cell subsets in the peripheral blood of the 11 participants (Fig. 4B). Interestingly, we were able to find intact provirus in all cell subsets studied, including SCM and TD. The total number of intact HIV-1 sequences ranged from 1 in SCM to 61 in EM (Table 3). Across all participants, there was no difference in intact infection frequency across cell subsets (*P* = 0.31, mixed-effects logistic regression), although EM was observed to have the highest intact infection frequency, followed by TM (Fig. 4B). Within each cell subset, there was no statistical evidence for a difference in the genetically-intact proviral infection frequency between the early and late participant groups (Table S2). This observation remained when time to ART initiation was treated as a continuous variable (Table S2).

## DISCUSSION

Understanding how cellular mechanisms such as proliferation and half-lives contribute to the genetic composition of HIV-1 within infected cells is critical for the development of effective curative strategies. We therefore performed a detailed genetic analysis of HIV-1 proviral DNA sequences derived from a broad range of peripheral blood CD4^+^ T-cell subsets with measured cellular half-lives from participants who initiated ART during early or late infection. Moreover, the measurement of these cellular half-lives allowed us to assess the relationship between total and genetically-intact HIV-1 provirus and the cellular half-life of the CD4^+^ T-cell subsets in the peripheral blood (28). Taken together, these studies characterize the extent to which cellular half-lives and proliferation of CD4^+^ T-cell subpopulations contribute to the persistence of genetically-intact and defective HIV-1 provirus.

We found that HIV-1-infected cells are more likely to have a shorter half-life (i.e., TM or EM cells) than a longer half-life (i.e., NV and CM), and we saw a similar trend toward enrichment of cells containing genetically-intact provirus in cells with a shorter half-life. The *in vivo* labeling of T-cell subsets with deuterated glucose in uninfected volunteers revealed that there are distinct T-cell subpopulations with differing turnover rates, and EM T-cells were three times more proliferative than CM T-cells (35). The results of this study as well as our previous results (24) showed that EM and TM cells, which have shorter half-lives, have high levels of HIV-1 provirus, providing further evidence for the enrichment of HIV-1 provirus in cells with a shorter half-life. Our previous results also showed that EM and TM cells have high levels of genetically-intact provirus (24), and although we did not see a statistical difference in this study, we did see a trend toward high levels of genetically-intact provirus in TM and EM cells. It is important to note that few genetically-intact sequences were isolated from each cell subset in this study, making it difficult to observe a statistically significant relationship between the level of genetically-intact provirus and cellular subset or cellular half-life. Therefore, our findings indicate that the short half-lives and rapid turnover rates of memory T-cells, such as TM and EM, contribute to the level of total HIV-1 provirus they contain. This agrees with Bacchus-Souffan et al. (28), who found cells with shorter half-lives contain more HIV-1 DNA by measuring integrated HIV-1 DNA. Since measurements of total or integrated HIV-1 DNA measure mostly defective proviruses (24, 36), it is important to assess the relationship between cellular half-lives and genetically-intact proviruses, as this will inform the field how specific cellular mechanisms, such as half-lives/turnover rates, maintain this replication-competent reservoir in HIV-1-infected participants during effective ART. Interestingly, we also find that the short half-lives of memory T-cells may contribute to the level of genetically-intact provirus detected.

Cellular proliferation, as evidenced by expansions of genetically-identical HIV-1 sequences, has been shown by multiple studies to contribute to HIV-1 persistence during ART (5, 6, 8, 9, 11, 13–18, 24, 25, 37). In our study, we found that the proportion of sequences belonging to an expansion of genetically-identical HIV-1 sequences was highest in the more differentiated cells (EM and TD). In addition, the integration site sequencing analysis in the larger study of 24 participants (28) found that more differentiated cells had higher levels of clonally expanded integration sites. The proliferative capacity of EM has been described earlier (24, 25, 38); however, the proliferative capacity of TD is still not fully understood. One study found the proliferative capacity of TD cells to be low (39), while other studies found evidence for high proliferative capacity of TD cells by detecting T-cell receptor clonality and the expression of Ki67 (38, 40). Our data support the latter studies, as here we find relatively higher levels of genetically-identical sequences in TD compared with the other subsets.

In our study, we found that 61% of all genetically-intact sequences were part of an expansion of identical sequences, suggesting that cellular proliferation plays an important role in maintaining this genetically-intact proviral population. Despite the high level of genetically-identical sequences in EM and TD, we only found genetically-intact sequences that belonged to an expansion of identical sequences or EIS in the TM and EM subsets, with relatively higher levels found in the EM subset. This supports our previous findings that the EM subset has the highest level of genetically-intact sequences that were part of an EIS (24). The proliferative potential of memory T-cells increases exponentially as cells transition from CM to EM cells (28). In primates, TM cells have a lower magnitude of expansion than EM cells in response to interleukin-15 (IL‐15) *in vivo* (41, 42). Therefore, the potential for highly proliferative EM cells containing genetically-intact provirus to undergo clonal expansion may explain why we see higher levels of genetically-intact proviruses in cells with shorter half-lives and more rapid turnover rates and why fewer intact proviruses are part of an EIS in TM than in EM. This enrichment within proliferating cells with short half-lives is demonstrated by studies showing that HIV-1 can be found in memory CD4^+^ T-cell clones that tend to be activated more often, leading to the proliferation of these infected cells (22). These T-cell clones include those that recognize chronic/persistent viral antigens such as cytomegalovirus (CMV) or HIV-1 (43–45). It is, however, important to mention that these genetically-identical intact proviruses were found in certain participants but not others. Larger studies with more participants would provide more statistical power to further investigate the role that cellular turnover rate plays in the enrichment of genetically-intact proviruses within specific T-cell subsets. This cellular proliferation inferred from EIS could also be explained by insertional mutagenesis in oncogenes, resulting in the enhanced proliferation of some clones (16, 46). However, Bacchus-Souffan et al. (28) showed that only 1.3% of integration sites in the studied participants were found either in, or close to, oncogenes. Altogether, from our data we propose a model showing that shorter cellular half-life could be a predictor of a higher frequency of genetically-intact provirus in cells.

We found genetically-intact sequences in all subsets studied. Similar to our previous findings (24), we observed EM and TM cells to have the highest infection frequency with genetically-intact provirus, although this difference was not statistically significant in this study. This may be due to the fact that the 11 participants included in this study have been on ART for a shorter amount of time (median, 3.5 years; Table 1) than those included in the previous study (median, 15.8 years) (24). It may also be due to the small number of genetically-intact proviruses isolated from each cell subset in this study. There are currently no detailed studies looking at genetically-intact provirus for two of the T-cell subsets included in this study: the progenitor-like SCM (47) and the differentiated but CD45RA-expressing TD cells (39). While the relative contribution of proviruses from SCM and TD cells to the overall HIV-1 reservoir is low, our results reveal that these cells can contain genetically-intact genomes. This suggests that HIV-1 exploits both the progenitor-like properties of SCM (47) and the proliferative capacity of TD (38, 40) to persist during therapy. Collectively, all cell subsets studied can contribute to HIV-1 persistence; however, the high level of genetically-intact provirus in EM and TM suggests that cellular mechanisms specific to these two T-cell subsets, such as a short half-life, contribute to viral persistence.

It will be interesting to see if the association between the half-life of a cell and the presence of genetically-intact provirus will hold true for CD4^+^ T-cell subsets that were not included in this study. For example, we have previously shown that HLA-DR^+^ cells contain high levels of genetically-intact HIV-1 (25). This cell subset has been found to have a very short half-life in HIV-negative participants (28), which agrees with our finding that cell subsets with shorter half-lives contain high levels of genetically-intact proviruses. However, our study only included resting HLA-DR^−^ CD4^+^ subpopulations, excluding events that occur during transient activation and HLA-DR expression of cells *in vivo*. Furthermore, a recent study has found clonally expanded, genetically-intact, and replication-competent provirus in functionally polarized Th1 cells in the peripheral blood, which were also not examined in our study (37). Lastly, all observations were restricted to circulating subpopulations of cells that are not representative of tissue-based cells, many of which do not recirculate. Less-characterized resident memory (T_RM_) T-cells are transcriptionally, phenotypically, and functionally distinct from recirculating memory cells and provide a first response against infections in tissue, where they accelerate pathogen clearance (48). The inclusion of HLA-DR^+^ cells, functionally polarized Th cells, and T_RM_ cells in future analysis will help expand our knowledge of how cellular mechanisms affect HIV-1 persistence.

The 11 participants in our study were a subset of the 24 participants in a recently published study characterizing CD4^+^ T-cell turnover and HIV-1 persistence as measured by integrated HIV-1 DNA, cell-associated HIV-1 RNA, and proviral integration site sequencing (28). The study by Bacchus-Souffan et al. (28) found that shorter cellular half-lives were associated with higher total integrated DNA and cell-associated RNA levels, which supports our findings that cell subsets with shorter half-lives, such as TM and EM, have higher levels of HIV-1 infection and genetically-intact provirus. For the 11 participants included in our study, we did not observe differences in the levels of HIV-1-positive cells or cells containing genetically-intact HIV-1 between the early and late ART treatment groups. In contrast, the study of 24 participants found the late ART group had higher levels of both integrated HIV-1 DNA and cell-associated RNA in CM, TM, and EM T-cell subsets. The limited number of participants in our study (11 versus 24) may explain this discrepancy. It is also important to note that Bacchus-Souffan et al. (28) did not include TD in their analysis because the TD subset was contaminated with NV cells during cell sorting. We decided to include the TD results here since we found high levels of genetically-identical provirus in the TD subset, and this is not characteristic of NV cells (9, 24, 25). However, our results may represent a mixture of TD and NV T-cells. For future analyses, it would be important to conduct these analyses on a pure TD subset.

It may be expected that cell populations with short half-lives, which are more activated and turn over rapidly, would express more virus and, therefore, be cleared by the immune response. However, our study revealed that the level of genetically-intact HIV-1 is higher in cells with a short half-life such as TM and EM cells. One plausible explanation for this observation is that these cell populations with short half-lives are also more likely to express viral proteins, such as Nef. The expression of the Nef protein in cells during therapy has been shown by several studies to contribute to the persistence of HIV-1 by downregulating cell-surface major histocompatibility complex class I (MHC-I) and antigen presentation, which then allows the virus to evade CD8^+^ T-cell clearance (49, 50). In agreement with these findings, a recent study has revealed that HIV-1-specific T-cell response, and, more specifically, HIV-1 Nef-specific response continues during long-term ART. These HIV-1 Nef-specific responses were associated with the frequency of HIV-1-infected cells and indicate recent *in vivo* recognition of the HIV-1 Nef antigen during ART (51). Therefore, viral protein expression in more activated cells with a short half-life/rapid turnover rate could protect genetically-intact genomes, which retain the ability to express protective viral proteins. It is therefore possible that the expression of viral proteins such as Nef from genetically-intact proviruses is contributing to immune evasion and the persistence of these proviruses within cells with a short half-life such as TM and EM populations.

There are several limitations to our study that deserve comment. First, we did not employ *in vitro* studies to confirm the replication-competency of intact provirus identified by FLIPS. Therefore, we cannot be certain that the genetically-intact proviruses we identified are truly replication-competent. Second, it is important to note that NV T-cells, due to their long half-life, may affect the results of the regression analysis. However, regression analysis that did not include NV T-cells still showed a significant correlation between cell half-life and HIV-1 proviruses. Third, we assume that HIV-1-infected cells have fractional replacement rate characteristics similar to those of uninfected cells of the same maturational phenotype, which has not been confirmed. Finally, there is a lack of inclusion of female participants in this study, and it is known that sex plays an important role in modulating T-cell biology (52, 53).

In summary, the distribution of HIV-1 genomes across T-cell subsets during ART suggests that a short cellular half-life could be a predictor of a higher frequency of total HIV-1 proviruses or genetically-intact proviruses. Furthermore, cell subsets with high levels of genetically-intact provirus were marked by clusters of genetically-identical HIV-1 genomes, reflecting cellular proliferation. This indicates that specific cellular mechanisms such as a relatively short half-life and greater proliferative potential, characteristics of EM T-cells, contribute to the maintenance of genetically-intact HIV-1. A more complete understanding of these cellular mechanisms may inform future HIV-1 curative strategies.

## MATERIALS AND METHODS

### Participant cohort.

Eleven participants were sampled from a larger recently published study (28) of 24 ART-suppressed subtype B HIV-infected individuals who underwent deuterated water labeling and large-volume blood draws for evaluation of resting CD4^+^ T-cell turnover within sorted subsets. Participant characteristics are available in Table 1. Participants were divided into two groups based on early (<6 months) or late (>6 months) ART initiation.

### *In vivo* labeling with deuterated (heavy) water.

Participants were provided with 70% deuterated water (^2^H_2_0), with an oral intake of 50 ml three times daily for the first 7 days and 50 ml twice per day for the remainder of the 45-day-long labeling period as recently described (28). Peripheral blood was collected at baseline and at days 15, 30, and 45. Plasma was collected at all four time points, and saliva samples were collected at days 7 and 21 during the first month for assessment of body water enrichment, which was used to calculate the cellular replacement rate (28).

### Cell sorting.

As previously described, fluorescence-activated cell sorting (FACSAria [BD Biosciences, Franklin Lakes, NJ, USA]) was used to sort resting (HLA-DR^−^) naive, stem-cell memory, central memory, transitional memory, effector memory, and terminally-differentiated CD4^+^ T-cell subsets (28). During the cell sorting, there was an estimated median of 20% contamination of the terminally-differentiated subset with naive cells based on an analysis of sjTREC content within each sorted population (28). Thus, the sorted terminally-differentiated population likely reflects a mix of naive and terminally-differentiated cells.

### Measurement of cellular half-lives using deuterium enrichment.

The stable isotope/mass spectrometric method for measuring cell proliferation has been described previously (54). The cellular half-lives were calculated by determining deuterium enrichment in cellular genomic DNA as measured by gas chromatography mass spectroscopy (28). Controls required for working with low cell count samples were included. The inferred half-lives reflect the rate at which cells of each subpopulation in the peripheral blood turn over, through cell birth or death, cellular maturation into other phenotypes, or trafficking to or from anatomic compartments. The half-life for each cell subset from each participant can be found in Table 2.

### FLIPS assay.

The FLIPS assay was performed as previously reported (24). Briefly, HIV-1 near-full-length proviruses (9 kb; 92% of the genome) were amplified at limiting dilution. Next-generation sequencing was conducted using the Illumina MiSeq platform. A median of 32 individual proviruses (range, 1 to 56) were sequenced per sorted CD4^+^ T-cell subset per participant (Table 3). Individual proviruses were assembled *de novo* using a specifically designed workflow in CLC Genomics (Qiagen). Proviruses were characterized as intact using a process of elimination that sequentially identified proviruses with inversions, large internal deletions (<8,900 bases), APOBEC3G-induced hypermutation, deleterious stop codons, frameshift mutations in any HIV-1 open reading frame (excluding *tat* exon 2 and *nef*) (55, 56), or a mutation in the packaging signal or MSD site (allowing for the presence of a cryptic MSD four nucleotides downstream of the MSD to salvage a mutated MSD [57]). Genomes were considered genetically-intact if they lacked such defects.

### Further analysis of sequences.

EIS were identified using ElimDupes (Los Alamos database; https://www.hiv.lanl.gov/content/sequence/elimdupesv2/elimdupes.html). All sequences that were 100% identical were considered part of an EIS cluster. Maximum likelihood phylogenetic trees using the generalized time-reversible model were estimated for each participant using FastTree version 2.1.12 (58). Branch support was inferred using 1,000 bootstrap replicates, and gaps/deletions were weighted by proportion of nongaps. Annotated tree images were constructed using the iTOL software, version 5.5.1 (59).

### Statistical analysis.

Statistical analysis was carried out in R version 3.6.3 (R Development Core Team, 2020). Mixed logistic regressions were carried out with the function *glmer* from the *lme4* package (60). Mixed logistic regressions were used for testing of binary endpoints (e.g., EIS and infection frequencies) to account for correlated sequence information within participants. In these regressions, a random effect for the intercept was always included. Random effects for cellular subset were also included for infection frequency, intact infection frequency, and expansions of identical sequences models based on evidence for effect modification between participant and cellular subset (*P* < 0.001). The pairwise comparison of cellular subsets was tested using function *glht* from the *multicomp* package (61) on the mixed logistic regression, which automatically adjusts *P* values for multiple comparisons using the Bonferroni method. To compare the proportion of intact (or defective) sequences that were part of an EIS across early and late groups, the sequences from each participant were pooled and a Fisher exact test was used. Here, a mixed logistic regression to account for correlated data within an individual was not possible due to the limited number of intact sequences. The association between cellular half-life and proviral load was assessed with a linear mixed-effect model with function *lmer* in package *lme4*. The half-life values were log transformed before all analyses were performed. A random effect was included for intercept only. Conditional and marginal *R*^2^ (c*R*^2^ and m*R*^2^, respectively) values were calculated using function *r.squaredGLMM* in package *MuMIn* (62). These *R*^2^ values are analogous to *R*^2^ correlation values and indicate the amount of variability in infection frequency that can be accounted for by cellular half-life. m*R*^2^ is the variability accounted for by the population-wide trend in half-life (Fig. 2, black line). The c*R*^2^ is the variability accounted for by the trend in half-life after adjusting for individual participant effects (Fig. 2, individual participant colored lines).

### Data availability.

The sequences analyzed in this study have been uploaded to GenBank under accession numbers MN466964 to MN467397, MZ922480 to MZ923010, and MZ962316.
