# Supplementary material for: HIV-1 Genomes Are Enriched in Memory CD4+ T-Cells with Short Half-Lives
Source: mBio. 2021 Sep 21;12(5):e02447-21. doi: 10.1128/mBio.02447-21 (PMC8546577; doi:10.1128/mBio.02447-21)
Supplement: TABLE S1 [file mbio.02447-21-st001.docx]

**Table S1. Linear regression analysis excluding NV T-cells.**

| **Analysis** | **ART Treatment Group** | **T-cell exclusion** | **Adjusted for cellular subset** | **P-value** |
| --- | --- | --- | --- | --- |
| Total infection frequency | All | NV T-cells | No | <0.001 |
| Total infection frequency | All | NV T-cells | Yes | 0.043 |
| Intact infection frequency | All | NV T-cells | No | 0.018 |
| Intact infection frequency | All | NV T-cells | Yes | 0.15 |
