# Supplementary figures and images for: HIV-1 Genomes Are Enriched in Memory CD4+ T-Cells with Short Half-Lives
Source: mBio. 2021 Sep 21;12(5):e02447-21. doi: 10.1128/mBio.02447-21 (PMC8546577; doi:10.1128/mBio.02447-21)

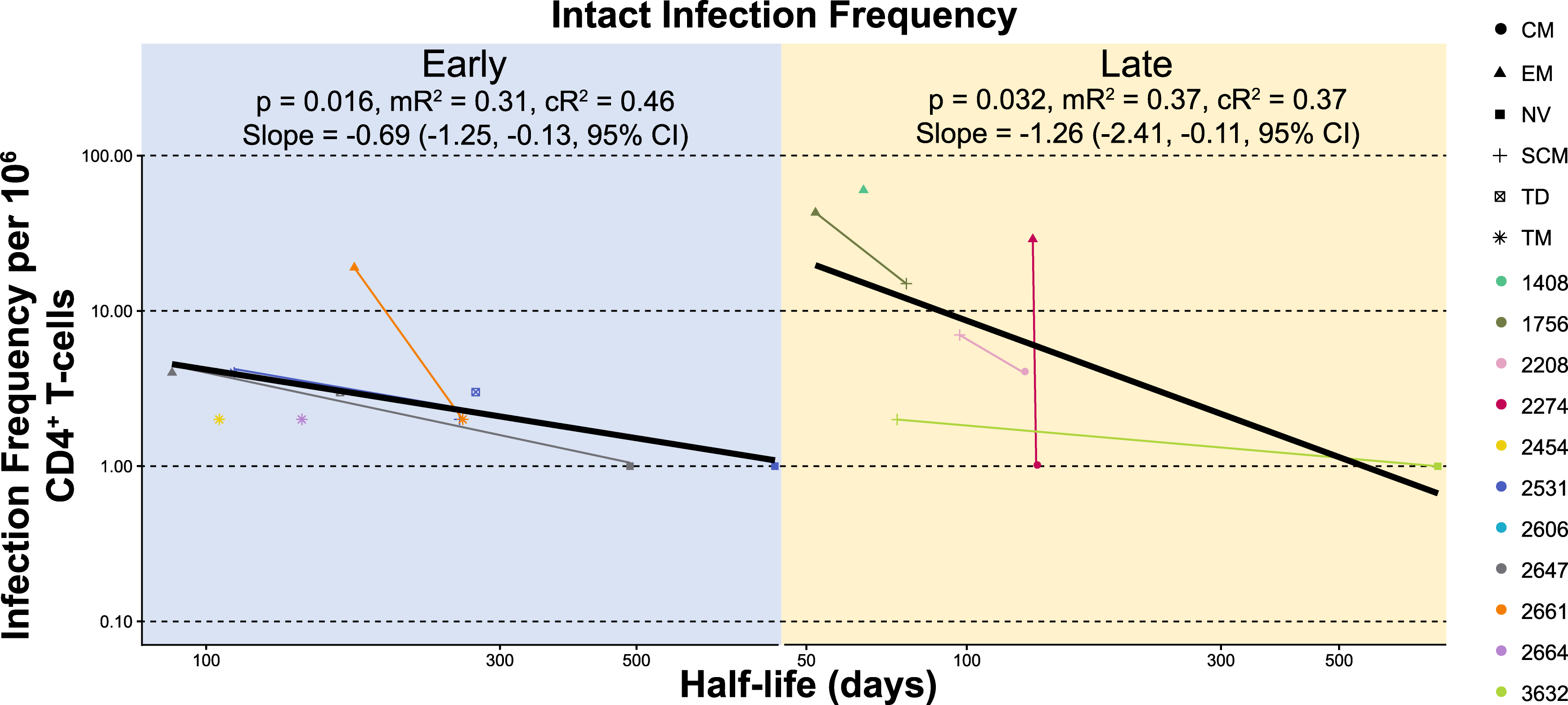

Supplement: FIG S1 [file mbio.02447-21-sf001.tif]

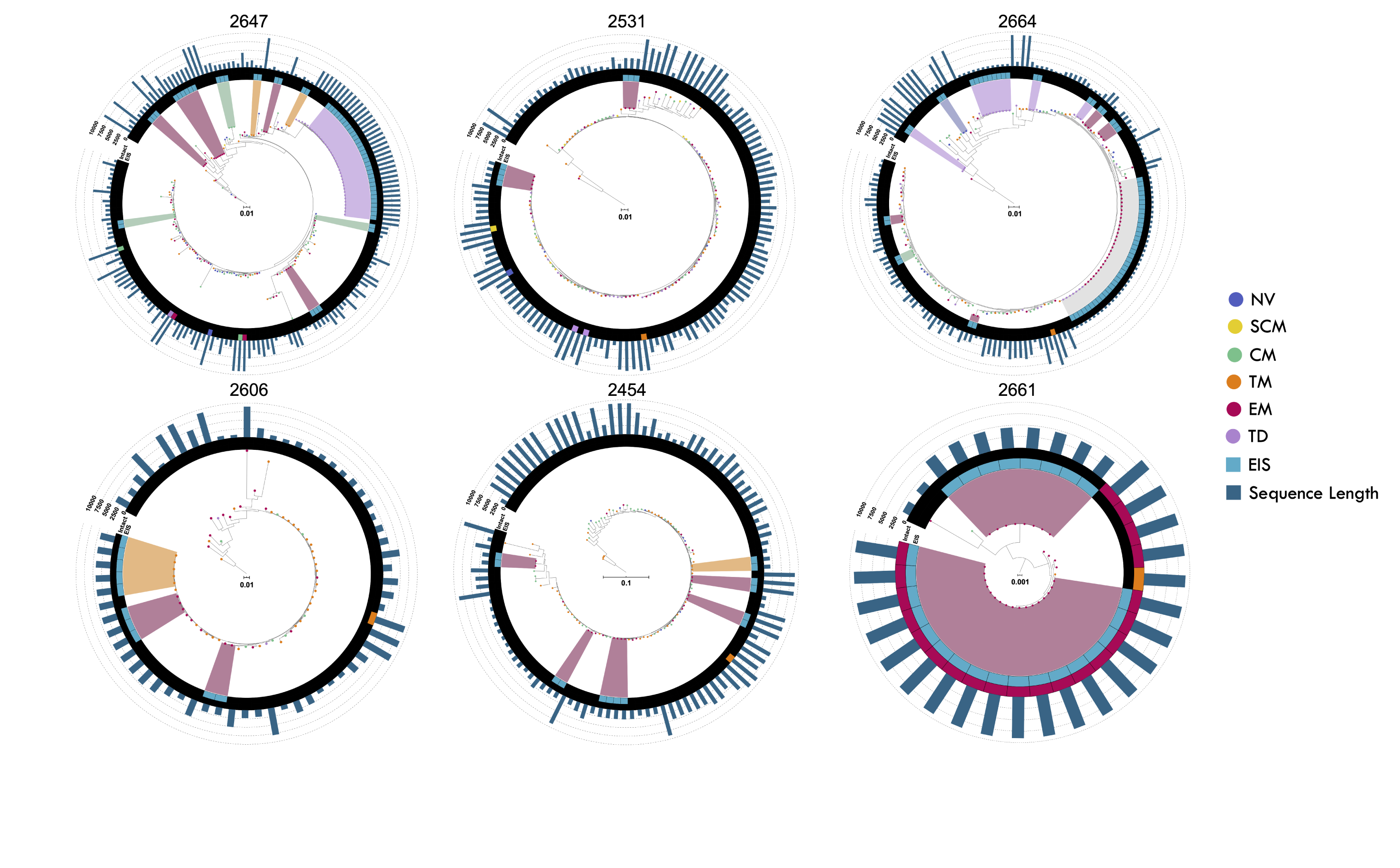

Supplement: FIG S2 [file mbio.02447-21-sf002.tif]

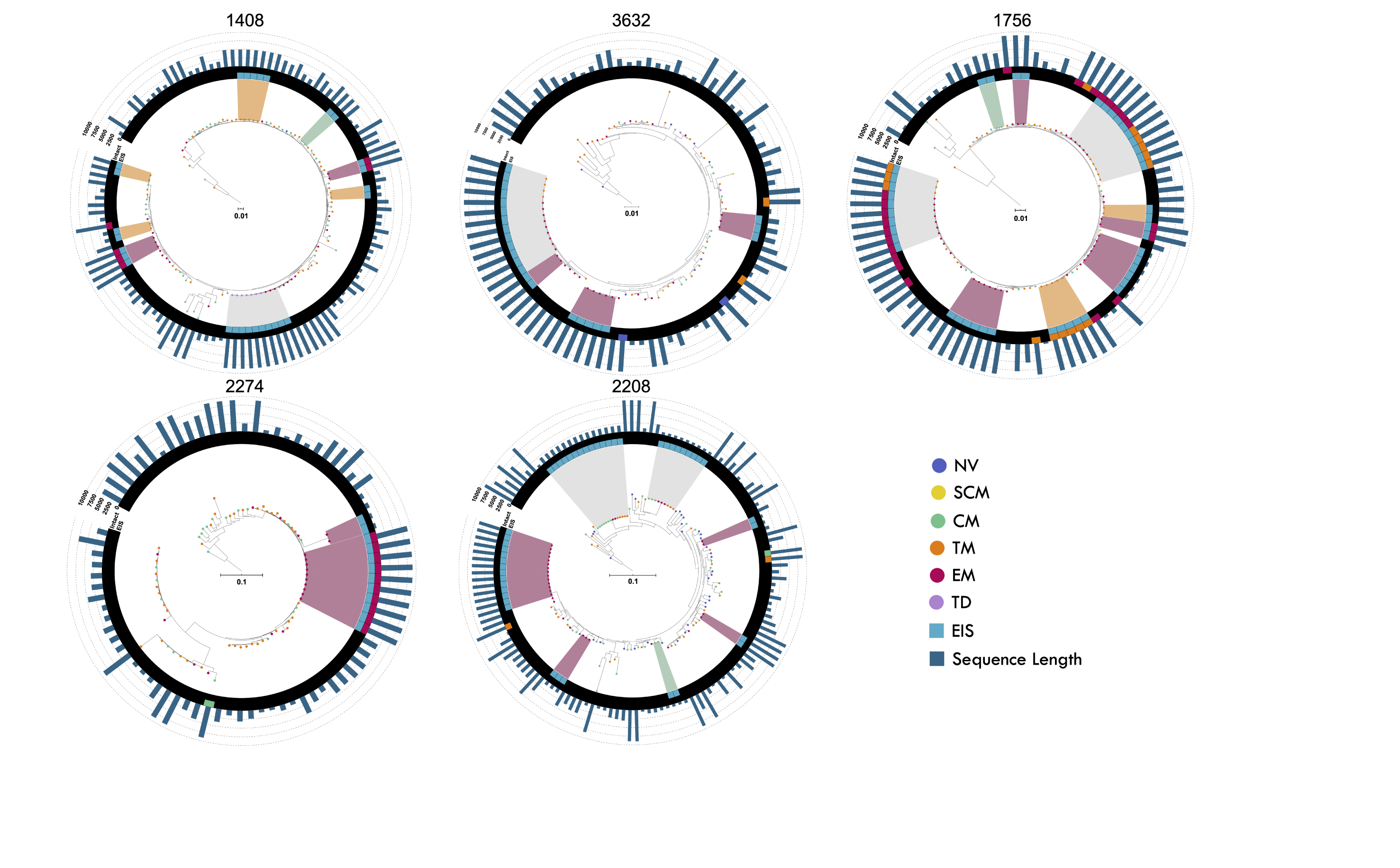

Supplement: FIG S3 [file mbio.02447-21-sf003.tif]
