# Supplementary material for: HIV-1 Genomes Are Enriched in Memory CD4+ T-Cells with Short Half-Lives
Source: mBio. 2021 Sep 21;12(5):e02447-21. doi: 10.1128/mBio.02447-21 (PMC8546577; doi:10.1128/mBio.02447-21)
Supplement: TABLE S2 [file mbio.02447-21-st002.docx]

**Table S2. Comparisons between Early and Late ART Treatment groups**

**(Mixed-effects logistics regression).**

| **Data** | **Subset** | **P-value** | **Comments** | **Time to ART initiation treated as continuous variable** | **Comments** |
| --- | --- | --- | --- | --- | --- |
| **Total Infection frequency** | NV | 0.80 |  | 0.10 | Early < Late |
|  | SCM | 0.24 |  | 0.24 |  |
|  | CM | 0.39 |  | 0.06 | Early < Late |
|  | TM | 0.49 |  | 0.18 |  |
|  | EM | 0.36 |  | 0.09 | Early < Late |
|  | TD | 0.09 | Early > Late | 0.20 |  |
| **Intact Infection Frequency** | NV | 0.97 |  | 0.70 |  |
|  | SCM | 0.99 |  | N/A |  |
|  | CM | 0.63 |  | 0.20 |  |
|  | TM | 0.18 |  | 0.44 |  |
|  | EM | 0.10 |  | 0.94 |  |
|  | TD | 0.99 |  | 0.48 |  |
| **EIS** | NV | 1 |  |  |  |
|  | SCM | Not enough data |  |  |  |
|  | CM | 0.16 |  |  |  |
|  | TM | <0.001 | Early < Late |  |  |
|  | EM | 0.25 |  |  |  |
|  | TD | 0.72 |  |  |  |
| **EIS and Intact** | NV | Not enough data |  |  |  |
|  | SCM | Not enough data |  |  |  |
|  | CM | Not enough data |  |  |  |
|  | TM | 1 |  |  |  |
|  | EM | 0.2 |  |  |  |
|  | TD | Not enough data |  |  |  |
